# Alteration of the Oligodendrocyte Lineage Varies According to the Systemic Inflammatory Stimulus in Animal Models That Mimic the Encephalopathy of Prematurity

**DOI:** 10.3389/fphys.2022.881674

**Published:** 2022-07-19

**Authors:** Geraldine Favrais, Cindy Bokobza, Elie Saliba, Sylvie Chalon, Pierre Gressens

**Affiliations:** ^1^ UMR 1253, iBrain, Inserm, Université de Tours, Tours, France; ^2^ Neonatology Unit, CHRU de Tours, Tours, France; ^3^ Inserm, NeuroDiderot, Université Paris Cité, Paris, France

**Keywords:** preterm infants, white matter, brain injuries, brain inflammation, experimental animal models

## Abstract

Preterm birth before the gestational age of 32 weeks is associated with the occurrence of specific white matter damage (WMD) that can compromise the neurological outcome. These white matter abnormalities are embedded in more global brain damage defining the encephalopathy of prematurity (EoP). A global reduction in white matter volume that corresponds to chronic diffuse WMD is the most frequent form in contemporary cohorts of very preterm infants. This WMD partly results from alterations of the oligodendrocyte (OL) lineage during the vulnerability window preceding the beginning of brain myelination. The occurrence of prenatal, perinatal and postnatal events in addition to preterm birth is related to the intensity of WMD. Systemic inflammation is widely recognised as a risk factor of WMD in humans and in animal models. This review reports the OL lineage alterations associated with the WMD observed in infants suffering from EoP and emphasizes the role of systemic inflammation in inducing these alterations. This issue is addressed through data on human tissue and imaging, and through neonatal animal models that use systemic inflammation to induce WMD. Interestingly, the OL lineage damage varies according to the inflammatory stimulus, i.e., the liposaccharide portion of the *E.Coli* membrane (LPS) or the proinflammatory cytokine Interleukin-1β (IL-1β). This discrepancy reveals multiple cellular pathways inducible by inflammation that result in EoP. Variable long-term consequences on the white matter morphology and functioning may be speculated upon according to the intensity of the inflammatory challenge. This hypothesis emerges from this review and requires further exploration.

## 1 Introduction

Preterm birth is a risk factor of neurodevelopmental delay. Improvements in prenatal and neonatal management have led to an increase in the survival of very preterm infants over time but have failed to reduce severe neurodevelopmental impairment which still concerns 10–15% of the surviving infants (Wilson-Costello et al., 2007; Moore et al., 2012; Pierrat et al., 2017). Moreover, an alteration in neurologic functions is present in about one third of the very preterm infants at 5 years old (Pierrat et al., 2021).

Brain imaging reveals that premature birth before the gestational age of 32 weeks is associated with specific white matter abnormalities which are visible in the neonatal period using ultrasound scans or at term-equivalent age through magnetic resonance imaging (MRI). Interestingly, this white matter damage (WMD) is associated with poorer motor and cognitive outcomes in very preterm infants (Woodward et al., 2006, 2012; Spittle et al., 2011; Campbell et al., 2021). Advances in brain imaging demonstrate that these white matter abnormalities are embedded in a more global brain damage defining the encephalopathy of prematurity (EoP) (Volpe, 2009; Hinojosa-Rodríguez et al., 2017).

The presence and severity of WMD are variable in each very preterm infant. The occurrence of prenatal, perinatal and postnatal events in addition to preterm birth may contribute to the intensity of the WMD (Korzeniewski et al., 2014; Barnett et al., 2018). Human and animal studies report that systemic inflammation and hypoxia-ischemia are major factors related to WMD in preterm infants (Khwaja and Volpe, 2007; Ophelders et al., 2020). The vulnerability of the cerebral white matter to ischemia is due to the poverty of arterial blood supply and the absence of cerebral blood flow regulation and protection during this brain developmental stage (Khwaja and Volpe, 2007). Epidemiological studies strongly support that perinatal infection/inflammation is related to WMD in very preterm infants (Wu, 2002; Shah et al., 2008; Procianoy and Silveira, 2012; Anblagan et al., 2016). Interestingly, Shah et al. reported that 40% of preterm infants with sepsis exhibited arterial hypotension (Shah et al., 2008). Therefore, although these factors can be considered separately, inflammation and hypoxia-ischemia could be closely intricated in the pathophysiology of EoP (Khwaja and Volpe, 2007; Volpe, 2008).

This review focuses on the OL lineage alterations associated with the WMD observed in infants suffering from EoP and emphasizes the role of systemic inflammation in inducing these alterations. This issue is addressed through data on human tissue and imaging, and through neonatal animal models that use systemic inflammation to induce WMD. Interestingly, the OL lineage damage varies according to the inflammatory stimulus, i.e., the liposaccharide portion of *E.Coli* membrane (LPS) or the proinflammatory cytokine Interleukin-1β (IL-1β). This discrepancy reveals multiple cellular pathways inducible by inflammation that result in EoP. This review also raises further questions about the long-term trajectories of OL lineage according to the inflammatory stimulus and the potential impact on brain structure and neurological functions.

## 2 White Matter Damage and Encephalopathy of Prematurity

### 2.1 White Matter Damage Observed in the Encephalopathy of Prematurity

Three patterns of WMD have been described in preterm infants from imaging data and autopsy series. The first pattern that was historically identified is periventricular leukomalacia. This form is characterised by multiple focal cysts that symmetrically surround lateral ventricles within the periventricular white matter. These cysts result from an intense and focal infiltration of macrophages and microglia exhibiting an amoeboid morphology that leads to tissue necrosis (Riddle et al., 2011). The disruption and degeneration of axons are observed within cysts (Riddle et al., 2012). Periventricular leukomalacia is associated with the occurrence of cerebral palsy in children (Bax et al., 2006). This severe morbidity is diagnosed in less than 4% of very preterm infants in contemporary cohorts from high-income countries (Ancel et al., 2015; Stoll et al., 2015). The second pattern is related to focal necrosis less than 1 mm in the deep periventricular white matter. These lesions progress to microcysts or to punctuate glial scars (Riddle et al., 2011; Buser et al., 2012; eurUS.brain group et al., 2020). The frequency of these non-hemorrhagic punctate lesions seems to decrease over time but the precise proportion is unknown, ranging from a few percent to 20% of preterm infants (Buser et al., 2012; Wagenaar et al., 2017; Parodi et al., 2019). The third form corresponds to the diffuse chronic WMD. This form includes a global reduction of white matter volume associated with ventriculomegaly, enlargement of the interhemispheric space and simplified cortical gyration. At the cellular level, large areas within the periventricular white matter are invaded by glial cells (Riddle et al., 2011; Buser et al., 2012). The recruitment of these glial cells differs according to the brain developmental stage. Microglial activation is predominant in the white matter of infants with a gestational age less than 32 weeks whereas intense astrogliosis is observed with a slight microglial activation in preterm infants with a gestational age from 32 to 36 weeks (Verney et al., 2012). Axons are preserved within the areas of gliosis (Riddle et al., 2012; Verney et al., 2012). While diffuse chronic WMD is currently the most frequent form, focal necrosis in the deep white matter can coexist with the diffuse form. The precise frequency of diffuse chronic WMD in very preterm infants is hard to determine as the intensity of this injury is variable in each infant. The human cohorts based on the neonatal brain imaging suggest that diffuse WMD could be present in about 40–70% of very preterm infants (Woodward et al., 2006; Kidokoro et al., 2013).

### 2.2 Alteration of the Oligodendrocyte Lineage and Encephalopathy of Prematurity

Myelin is visible in the human brain from 30 weeks of gestation and increases thereafter (Hüppi et al., 1998; Mukherjee et al., 2001; Bobba et al., 2022). However, oligodendrocytes (OLs) appear earlier in the developing brain (van Tilborg et al., 2018). Four successive stages are usually distinguished according to specific markers: 1) the oligodendrocyte precursor cells (OPCs) appear from neural stem cells. These cells successively come from specific areas of the ventricular neuroepithelium during brain development, i.e., from the medial ganglionic eminence, then, from the lateral ganglionic eminence and lastly, from the dorsal subventricular zone around birth in rodents (van Tilborg et al., 2018). Then, OPCs which show positive staining for PDGF-αR and NG2 antibodies migrate throughout the brain; 2) once OPCs reach their programmed and final destination, they evolve to premyelinating oligodendrocytes (Pre-OLs) that correspond to the last proliferative OL stage. Pre-OLs are characterised by positive staining for NG2 and O4 and negative staining for O1; 3) OLs then differentiate in order to become non-proliferative OLs capable of generating myelin. Immature OLs are the first OL stage after OL differentiation. Immature OLs are positive for CNPase and O1 staining; and 4) mature OLs produce myelin and wrap axons to form the myelin sheath. Mature OLs are positive for CNPase, APC (or CC-1) antibodies and the staining of myelin proteins such as the Myelin-Basic-Protein (MBP) (Salmaso et al., 2014; van Tilborg et al., 2018).

Pre-OLs play a pivotal role in the physiopathology of EoP. In mammals, WMD only appears when insults occur at the peak of the Pre-OL stage, i.e., around the embryonic day 25 in rabbits, around the gestational day 105 (70% of gestation) in sheep, around the postnatal day 2 (PND2) in rodents, and from 24 to 32 weeks of gestation in humans (Back et al., 2001; Dean et al., 2011; Buser et al., 2012; Salmaso et al., 2014; Galinsky et al., 2020). Excessive and specific mortality of Pre-OLs is observed in experimental conditions that reproduce the hallmarks of EoP. Two distinct and successive waves of Pre-OL death are described. An early death of Pre-OLs results from an alternative pathway to apoptosis, i.e., negative for cleaved-caspase-3 labelling. A late Pre-OL death is observed with a positive staining for the cleaved-caspase-3 antibody, arguing for Pre-OL apoptosis (Segovia et al., 2008; Riddle et al., 2011). Pre-OLs exhibit specific characteristics that make them vulnerable to cell death mechanisms. *In vitro*, Pre-OLs are more vulnerable to oxidative stress than other OL stages. Pre-OLs show low levels of glutathione which is involved in the antioxidant cell defence (Back et al., 1998). This deficit promotes lipid peroxidation through the 12-lipoxygenase activation and reactive oxygen species accumulation (Wang et al., 2004; Back et al., 2005). In parallel, Pre-OL apoptosis is observed in the presence of the pro-inflammatory cytokine Tumor Necrosis Factor-alpha (TNF-α) in the extra-cellular space (Pang et al., 2010; Su et al., 2011; Wang et al., 2014). It was suggested that early Pre-OL death is related to oxidative stress and late Pre-OL death rather than to the TNF-α-induced apoptosis (Pang et al., 2010). At the same time, an increase in Pre-OLs is observedwithin the large areas of gliosis (Buser et al., 2012). In contrast, immature and mature OL populations decrease. The global OL population is preserved without any change in OL proliferation or mortality. This imbalance in the OL lineage is due to a transient arrest of the OL lineage progression beyond the Pre-OL stage (Segovia et al., 2008; Favrais et al., 2011). This failure in OL differentiation results in a delayed myelination and in diffuse chronic WMD. This disruption of OL differentiation is considered to be the predominant mechanism in the contemporary cases of EoP (Riddle et al., 2011).

### 2.3 Inflammation is a Risk Factor of WMD in Preterm Infants

Several inflammatory challenges can occur over the perinatal period of preterm infants. During the third trimester of pregnancy, chorioamnionitis combines a possible fetal exposure to a bacterial agent, immune cell infiltration of the umbilical cord and a fetal inflammatory response with an increase in proinflammatory cytokines in fetal blood (Dammann et al., 2002; Jain et al., 2022) (Jain et al., 2022). Moreover, chorioamnionitis is associated with extremely preterm births and with an increase in neonatal morbidities such as neonatal sepsis (Pappas et al., 2014; Venkatesh et al., 2019; Beck et al., 2021). Although clinical chorioamnionitis is constantly associated with a worse neurodevelopmental outcome and periventricular leukomalacia in preterm infants, the impact of histological chorioamnionitis on neurological issues is controversial (Wu, 2002; Pappas et al., 2014; Bierstone et al., 2018; Maisonneuve et al., 2020; Konzett et al., 2021). During the postnatal period, an increase in proinflammatory cytokines is observed in the blood of infants suffering from necrotizing enterocolitis and postnatal sepsis (Sharma et al., 2007). This increase is much stronger in the case of endotoxin release in the blood (Sharma et al., 2007). Interestingly, the presence of WMD at term-equivalent age in preterm infants has been steadily associated with neonatal morbidities such as postnatal sepsis, necrotizing enterocolitis and bronchopulmonary dysplasia (Shah et al., 2008; Gagliardi et al., 2009; Shim et al., 2012; Barnett et al., 2018; Glass et al., 2018; Dubner et al., 2019). Furthermore, the cytokine increase in the preterm infant blood due to sepsis is independently associated with WMD (Procianoy and Silveira, 2012). Some authors therefore argue that WMD results from the repetition of systemic inflammatory challenges over the perinatal course (Shim et al., 2012; Korzeniewski et al., 2014; Barnett et al., 2018; Glass et al., 2018; Dubner et al., 2019).

Several animal models show that systemic infectious/inflammatory insults lead to white matter injuries of the developing brain. *Escherichia Coli*, a Gram-negative bacterium, was first used through intraperitoneal injections in kittens (Gilles et al., 1977). These injections resulted in cysts within the periventricular white matter (Gilles et al., 1977). Thereafter, the liposaccharide portion of the *Escherichia Coli* membrane (LPS), which is more stable than the entire bacterium, has been widely used in animal models to mimic bacterial endotoxemia (Wang et al., 2006). Depending on the experimental schedule, LPS can trigger hemodynamic alterations that could contribute to cerebral effects. A bolus of high-dose LPS induces immediate and transient hypotension, heart rate variability, decrease in cerebral oxygen delivery and sometimes mortality in preterm fetal sheep (Dalitz, 2003; Mathai et al., 2013). This challenge results in periventricular cystic necrosis (Mathai et al., 2013). Intriguingly, LPS mostly leads to periventricular WMD with the preservation of cortical grey matter whereas a pure ischemic-reperfusion insult in fetal sheep induces cortical and white matter injuries (Mallard et al., 2003)**.** Conversely, prolonged exposure to low doses of LPS is not associated with such hemodynamic changes but always promotes a strong systemic inflammatory response and microglial activation (Duncan et al., 2006; Keogh et al., 2012; Mathai et al., 2013). Diffuse hypomyelination resulted from this experimental schedule (Galinsky et al., 2020). Dammann et al. argued that the pro-inflammatory cytokine release in fetal blood is the link between the bacterial colonisation of placenta and WMD in the fetus in the chorioamnionitis context (Dammann et al., 2002). A purer and lesser inflammatory injury consisting of intraperitoneal injections of Interleukin-1β (IL-1β) to neonatal mice from PND1 to PND5 was performed to explore this hypothesis (Favrais et al., 2011; Van Steenwinckel et al., 2019). This experimental schedule increased IL-1β and TNF-α in the pup blood. Although the ventilation minute was slightly reduced, the heart rate, blood partial pressure in oxygen and cerebral blood flow were not altered by systemic IL-1β. Interestingly, this neonatal exposure to IL-1β led to a diffuse hypomyelination at the PND 30 (Favrais et al., 2011).

Therefore, systemic inflammation is widely recognised as a risk factor of WMD in humans and mammal models. Systemic inflammation leads to the complete range of preterm WM injuries from periventricular leukomalacia to diffuse chronic WMD in animal models. The resulting injury within the white matter depends on the inflammatory stimulus and the experimental schedule. Various cellular pathways can be activated according to the type of systemic inflammation (Table 1). In the section below, we focus on the underlying mechanisms that alter OL lineage in models using systemic LPS or IL-1β to induce diffuse hypomyelination.

**TABLE 1:** Cellular pathways that are implicated in the signalling of systemic LPS and systemic IL-1β that impair the OL lineage in the developing brain.

	Early Pre-OL Death	Early Impairment of OL Proliferation	Late Improvement of OPC Proliferation	Late Enhancement of OL Differentiation	Late Support of the Mature OL Survival	Alteration of OL Differentiation
Systemic LPS						
Microglia						
NF-κB pathway	—	—	—	—	—	—
↑ TNF-α	•	•	—	—	—	—
↑ IL-1β	—	•	•	•	•	—
↑ NO/ROS	•	—	—	—	—	—
Oligodendrocyte						
Oxidative stress	—	—	—	—	—	—
Glutathion deficit	•	—	—	—	—	—
12-α- lipoxygenase	•	—	—	—	—	—
TNF-α-induced cell-death pathways	—	—	—	—	—	—
**↑** RIP-1	•	—	—	—	—	—
MAPK pathways	—	—	—	—	—	—
p-JNK	•	•	—	—	—	—
p-ERK	•	•	—	—	—	—
p38	—	—	—	•	•	—
Systemic IL-1β						
Microglia						
Wnt/β-Catenin	—	—	—	—	—	•
COX-2	—	—	—	—	—	—
**↑** PGE2	—	—	—	—	—	•
Oligodendrocyte						
Wnt/β-Catenin	—	—	—	—	—	—
↑Axin 2 mRNA	—	—	—	—	—	•
↑Tcf4 mRNA	—	—	—	—	—	•
Unbalance of transcriptional factors that drive OL differentiation					
↑ SOX10 mRNA	—	—	—	—	—	•
↓ SOX 8 mRNA	—	—	—	—	—	•
↑ Olig1 mRNA	—	—	—	—	—	•
↓ Olig2 mRNA	—	—	—	—	—	•
↓ Nkx2.2. mRNA	—	—	—	—	—	•

## 3 Oligodendrocyte Lineage Response to Various Systemic Inflammation Stimuli in Animal Models that Mimic Encephalopathy of Prematurity

**FIGURE 1:**
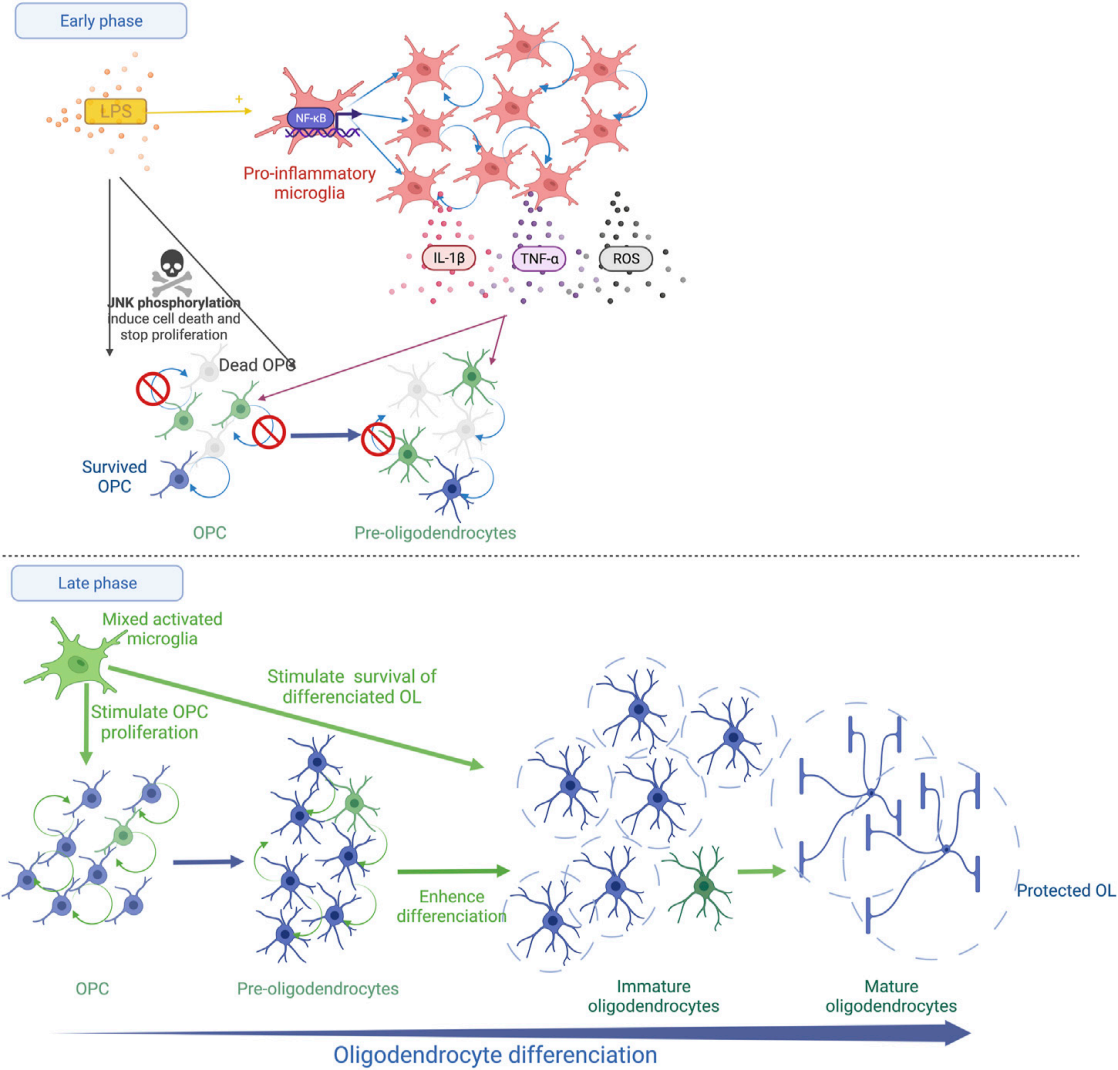
Schematic representation of systemic LPS action on OL lineage in the developing brain. The immediate LPS effect consists in the recruitment and the activation of microglia, which releases pro-inflammatory cytokines (TNF-α and IL-1β) and reactive oxygen species (ROS). This strong microglial activation leads to OPC/Pre-OL death and a reduction in OL proliferation (Early phase). Thereafter, a recovery phase occurs and microglia show a trophic phenotype. The proliferation of OPCs/Pre-OLs, the differentiation of OLs and the survival of mature OLs are promoted (Late phase).

**FIGURE 2:**
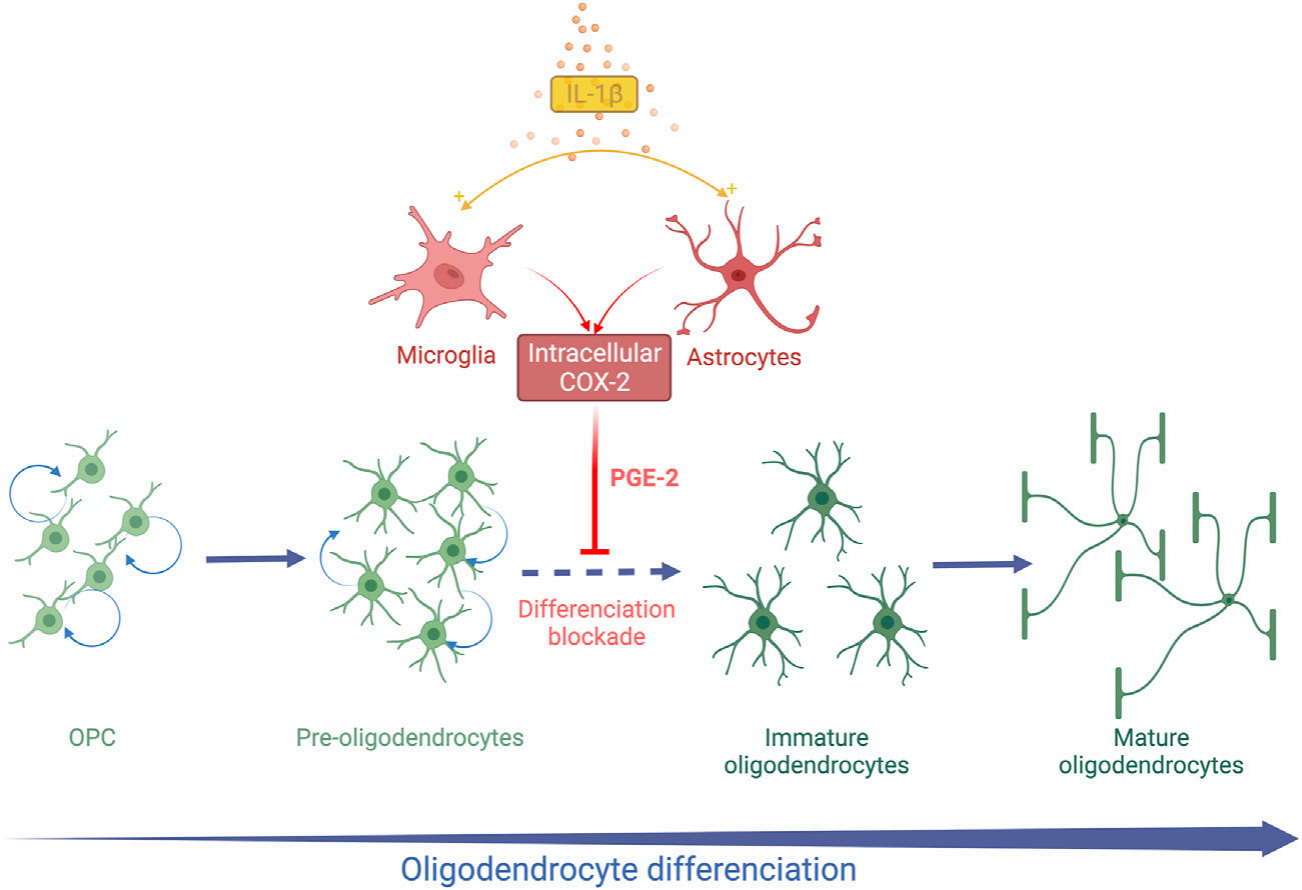
Schematic representation of systemic IL-1β action on OL lineage in the developing brain. Systemic Il-1β acts through the activation of the cyclooxygenase 2 (COX2)-Prostaglandin E2 (PGE2) pathway in astrocytes and microglia. PGE2 induces an arrest of OL differentiation leading to an accumulation of Pre-OLs and a reduction in differentiated OLs.

Animal models demonstrate that systemic inflammation during the vulnerability window of EoP can induce diffuse periventricular hypomyelination (see *Inflammation is a Risk Factor of WMD in Preterm Infants Section*). The OL lineage alterations that result in hypomyelination have been previously described in animal models using systemic LPS or systemic IL-1β. In the following section, these disturbances of OL lineage are reported as well as the cellular pathways activated according to each of these inflammatory stimuli (Table 1; Figures 1, 2).

### 3.1 Animal Models Using Systemic LPS During Late Pregnancy or Early Neonatal Life

#### 3.1.1 Experimental Models

LPS is an extract of the *Escherichia Coli* membrane that induces immune and inflammatory responses due to a bacterial stimulus. The usual way to induce a systemic inflammatory response by LPS is intraperitoneal injection either to neonatal rats or to adult rats during late pregnancy. Various experimental schedules are used in neonatal rats including a single 1 mg/kg dose of LPS, i.e., about 5–10 μg, at PND1 or at PND3, or a single 2 mg/kg dose of LPS, i.e., about 15–20 μg, at PND5 (Wong et al., 2014; Pang et al., 2016; Xie et al., 2016). LPS can also be injected to pregnant rats at gestational day 19 and 20 at the daily dose of 300 μg/kg, i.e. about 100 ug, to mimic the chorioamnionitis context (Rousset et al., 2006). In preterm fetal sheep, catheterization of the femoral vein enables continuous infusion of LPS (100–3200 ng/kg per day) and repetitive 1-ug boluses for several days (Mathai et al., 2013; Magawa et al., 2022).

#### 3.1.2 Effects on Oligodendrocyte Lineage

After LPS exposure during late fetal life in rats, the density of MBP staining is reduced within the external capsule at PND7 indicating diffuse periventricular hypomyelination (Rousset et al., 2006; Favrais et al., 2011).The density of myelin is completely restored at PND21 with a trend to a more intense MBP staining in the LPS group than in the control group (Favrais et al., 2021). In this model, the early stages of oligodendrocyte lineage that are positive for the NG2 staining decrease within the external capsule 48 h after the LPS challenge. The proliferative OL stages are also reduced within the periventricular white matter early after a neonatal LPS challenge in rats (Wong et al., 2014; Xie et al., 2016). The presence of pyknotic cells positive for the O4 staining was reported 24 h after the LPS injection, evoking a Pre-OL death (Wong et al., 2014). This deficit is transient. A rebound of the proliferative stage of OL lineage is observed a few days later (Pang et al., 2016; Xie et al., 2016; Favrais et al., 2021). Then, roughly two weeks later, the LPS exposure leads to an increase in the differentiated OLs, i.e, immature and mature OLs, within the white matter in neonatal rats and fetal sheep (Pang et al., 2016; Galinsky et al., 2020; Favrais et al., 2021). These results suggest that LPS induces an immediate Pre-OL death and/or a reduction in Pre-OL proliferation followed by an overproliferation and a sustained enhancement of OL differentiation. Intriguingly, LPS initiates an unexpected late phase that consists of trophic and protective effects of the differentiated OLs (Pang et al., 2016) (Figure 1).

#### 3.1.3 Implicated Mechanisms

LPS acts through the Toll Like Receptor 4 (TLR4) signalling. Various cellular pathways are downstream to TLR4 including activation of the NF-kB and the mitogen-activated protein kinase (MAPK) pathways. The NF-κB pathway strongly induces the release of pro-inflammatory cytokines such as IL-1β through the NLRP3 inflammasome and TNF-α (Yao et al., 2013; Zusso et al., 2019). The MAPK pathways including the p38, extracellular signal-regulated kinase (ERK) and c-Jun-N-terminal kinase (JNK) signalling are implicated in the regulation of OL proliferation and differentiation (Chew et al., 2010). TLR4 is present in the surface of microglia and its presence is suggested on OLs (Taylor et al., 2010; He et al., 2021).

Microglia are a key cells in the neonatal brain response to LPS. LPS induces microglia proliferation and activation with a rapid progression to an amoeboid shape within the white matter (He et al., 2021). LPS strongly initiates TNF-α release in the culture medium by microglia with a peak 24 h after the LPS challenge (Miller et al., 2007). TNF-α immunoreactivity also soars *in vivo* in the fetal sheep brain after LPS exposure (Mathai et al., 2013). IL-1β is also secreted by the LPS-activated microglia with a later peak and for a longer time (Taylor et al., 2010; He et al., 2021). Microglia also induce the production of nitric oxide and reactive oxygen species (ROS) through TLR4 activation (Yao et al., 2013).

Interestingly, the LPS-activated microglia show various effects on OLs depending on their developmental stage *in vitro* (Miller et al., 2007). A reduction in OPC/Pre-OL survival is observed in the presence of microglia activated by LPS according to two successive mechanisms (Miller et al., 2007; Pang et al., 2010). Oxidative stress induces an early death of OPCs/Pre-OLs (Pang et al., 2010). The arachidonic acid-induced ROS up-regulates the receptor interacting protein-1 (RIP-1) which triggers necroptosis, the regulated form of necrosis, in Pre-OLs depleted in glutathion (Kim et al., 2010). Then, a later death occurs mediated by the microglia-secreted TNF-α (Pang et al., 2010). Through its receptor 1 (TNFR1), TNF-α initiates three cell death pathways, i.e., 1) necroptosis, 2) RIP-1-dependent apoptosis and 3) RIP-1-independent apoptosis linked to the TNFR-associated death domain (TRADD) (Cao and Mu, 2021). Interestingly, an inhibitor of RIP-1/RIP-3 prevents OPC apoptosis following a hypoxic stimulus in the neonatal rat brain (Zhang et al., 2021). The neutralisation of TNF-α in the culture medium of LPS-activated microglia partially prevents OPC/Pre-OL death (Pang et al., 2010; Taylor et al., 2010). In parallel, an alteration of OPC/Pre-OL proliferation is also observed in the presence of microglia activated by LPS *in vitro* and after systemic LPS in neonatal rats (Taylor et al., 2010; Xie et al., 2016). Interestingly, the addition of IL-1β to the medium of OL culture without microglia inhibits Pre-OL proliferation without promoting OL death (Vela, 2002; Xie et al., 2016). In contrast, the LPS-activated microglia sustain the survival of mature OLs *in vitro*, consistent with the *in vivo* findings (Miller et al., 2007; Taylor et al., 2010; Pang et al., 2016; Favrais et al., 2021). Similar effects are observed by adding IL-1β to the medium of differentiated OLs *in vitro* (Vela, 2002). Pang et al. showed that systemic LPS in neonatal rats activates microglia in a brain region-specific manner 3 days after the LPS injection toward its pro-inflammatory M1 phenotype but also toward its M2 phenotype that promotes cell survival and proliferation (Pang et al., 2016). Proliferative cells are still detected 18 days after the LPS injection (Pang et al., 2016). Furthermore, LPS exposure during the neonatal period induces a long-lasting preconditioning effect on microglia that promotes OPC proliferation, mature OLs and remyelination in adulthood after cuprizone-induced demyelination (Skripuletz et al., 2011; Bénardais et al., 2014) (Figure 1).

Regarding OLs, the LPS signalling emphasizes the MAPK pathways. JNK phosphorylation results in Pre-OL apoptosis *in vivo* (Wang et al., 2012, 2014). Conversely, the suppression of the JNK1 pathway induces an overproliferation of OPCs with an alteration of OL branching and a myelination deficit (Lorenzati et al., 2021). TNF-α through its receptor TNFR1 induces JNK phosphorylation in OLs (Wang et al., 2014). In parallel, LPS can directly elicit JNK phosphorylation in OPCs/Pre-OLs in the absence of microglia *in vitro* (Taylor et al., 2010). ERK activity is also predominant in the early OL stages (Horiuchi et al., 2006; Chew et al., 2010). The suppression of ERK signalling is required to initiate OL differentiation (Chew et al., 2010). In inflammatory conditions, activation of the ERK pathway is associated with Pre-OL death and with a decrease in OPCs/Pre-OL proliferation (Horiuchi et al., 2006). In contrast to ERK expression, p38 MAPK is mainly expressed in the differentiated OLs. The p38 MAPK pathway sustains the OL differentiation process and possibly plays a role in myelin maintenance *in vivo* (Chew et al., 2010). Interestingly, the p38 MAPK pathway is up-regulated in OLs by exogenous IL-1β *in vitro* to promote their differentiation and the survival of mature OLs (Vela, 2002).

Therefore, LPS induces complex and long-lasting effects on OL lineage. The immediate phase elicits Pre-OL death and an alteration of Pre-OL proliferation. In a late phase, a trophic effect promotes OPc proliferation, OL differentiation and the survival of the differentiated OL stages, long after the LPS stimulus. The microglia response to LPS is likely to drive this biphasic kinetic through the regulation of oxidative stress and cytokine expression (Figure 1).

### 3.2 Mouse Model Using Systemic IL-1β During the Neonatal Period

#### 3.2.1 Experimental Model

This model is based on a more prolonged and modest systemic inflammation than that induced by LPS. Neonatal mice were intraperitoneally injected with 40 ng of IL-1β twice a day from PND1 to PND5. Microglia cells transiently increased up to PND5 (Favrais et al., 2007). Although a decrease of microglia arborization was observed at PND3 in mice exposed to IL-1β, microglial cells showed a similar density of processes and the same area covered by these processes as in control mice (Van Steenwinckel et al., 2019). The expression profiles of M1 and M2 markers by microglia during the IL-1β schedule were studied. The mRNA expression of pro-inflammatory (M1) markers soared rapidly after the first IL-1β injection and decreased thereafter. Some anti-inflammatory (M2) markers such as Arginine 1 transiently increased at PND2 and PND3 during IL1-β exposure. The expression levels of all markers were similar to those of the control group at PND10 (Van Steenwinckel et al., 2019). These results highlight that the IL-1β schedule creates a more transient and less intense microglia activation than LPS.

#### 3.2.2 Effects on Oligodendrocyte Lineage

A decrease in the MBP staining density is observed within the white matter at PND 15 and PND30 in the mice exposed to IL-1β during their neonatal period (Favrais et al., 2011). The PND35 mice exposed to IL-1β show the same white matter characteristics on *ex-vivo* brain MRI as those observed in preterm infants suffering from diffuse chronic WMD (Favrais et al., 2011). This deficit in myelin is transient as the myelin density in the IL-1β mice becomes similar to the control mice at PND60 (Favrais et al., 2011). This diffuse hypomyelination is associated with an early increase in the OPC/Pre-OL population from PND5 to PND15. In parallel, immature and mature OLs are reduced within the external capsule at PND15 and PND30. This alteration of OL lineage is not associated with modification of cell proliferation and apoptosis (Favrais et al., 2011). These data argue that neonatal systemic IL1-β induces an arrest of OL lineage differentiation from Pre-OL to immature OL stages (Figure 2).

#### 3.2.3 Implicated Mechanisms

The differentiation of OLs from proliferative stages to post-mitotic stages is linked to a complex balance between transcription factors such as Olig1, Olig2, Nkx2.2, SOX10, Axin2, Tcf4 (Stolt et al., 2002; Fu et al., 2009; Chew et al., 2010; Fancy et al., 2011; Dai et al., 2015; Wegener et al., 2015; Zhang et al., 2020). The mRNA expression of these transcription factors is modified up to PND15 in the brain of mice treated with IL-1β, supporting the hypothesis that IL-1β disrupts OL differentiation (Favrais et al., 2011) (Table 1).

The canonical Wnt/β-catenin signalling is likely to play a critical role in the alteration of OL differentiation. Tcf4 and Axin2 are downstream to the Wnt pathway and interact with *β*-catenin. These two transcription factors have been shown to be crucial to ensure the OL differentiation process (Fu et al., 2009; Fancy et al., 2011). The specific dysregulation of the Wnt/β-catenin pathway in the OL lineage in the developing brain disrupts OL differentiation (Fancy et al., 2009). Moreover, the targeted inhibition of Wnt/β-catenin signalling within microglia prevents IL-1β-induced hypomyelination (Van Steenwinckel et al., 2019).

Cyclooxygenase 2 (COX2) is an isoform of cyclooxygenase inducible by inflammation and initiates the synthesis of prostaglandin E2 (PGE2) from arachidonic acid. In contrast, the cyclooxygenase isoform 1 (COX1) is constitutionally expressed. Astrocytes and microglia are able to express COX2 in the human fetal brain during the third trimester of pregnancy (Shiow et al., 2017). The COX2- PGE2 pathway was therefore explored in this model of EoP. COX2 expression was upregulated in microglia and astrocytes after neonatal exposure to IL-1β (Shiow et al., 2017). In parallel, the amount of PGE2 increased in the brain of IL-1β mice at PND5, arguing for the activation of the COX2-PGE2 pathway by systemic IL-1β (Favrais et al., 2007) (Figure 2). As the four receptors of PGE2 (EP-1 to EP-4) are expressed by OLs, the direct action of PGE2 on OLs *in vitro* was explored. PGE2 induced a decrease in MBP-positive OLs in a dose-dependent manner *in vitro*. An increase in OPCs was also observed without any modification of cell proliferation or death as *in vivo*. Furthermore, Nkx2.2. expression was altered in OLs exposed to PGE2, supporting an alteration of OL differentiation (Shiow et al., 2017). Genetic and pharmacological neutralization of the EP-1 receptor prevented the deleterious effect of PGE2 on OL lineage. Then, mice exposed to IL-1β were also treated with nimesulid, a nonsteroidal anti-inflammatory drug (NSAID) and a specific inhibitor of COX2 from PND1 to PND5. Interestingly, nimesulid prevented IL-1β-induced hypomyelination and OL lineage alteration *in vivo* (Favrais et al., 2007; Shiow et al., 2017). Therefore, the COX2-PGE2 pathway could be a target for neuroprotective strategies in this context. Indomethacin, a COX1 and COX2 inhibitor, is routinely used for treating persistent ductus arteriosus in preterm infants. Two observational clinical studies showed a reduction of white matter injuries in very preterm infants who were sustainably treated with indomethacin (Miller et al., 2006; Gano et al., 2015). Although these results support our experimental hypothesis, large randomized controlled trials are needed to confirm these observations. However, NSAIDs show side-effects in preterm infants such as gastrointestinal haemorrhage and renal failure that could limit their widespread use for neuroprotection (Ohlsson and Shah, 2020).

Therefore, the exposure to systemic IL-1β during the neonatal period induces a transient arrest of OL differentiation. Microglia and astrocytes are likely to play a pivotal role in the systemic-IL-1β effects on OL lineage (Figure 2). The IL-1β model mimicked the OL lineage alteration observed in diffuse chronic WMD within the gliosis areas.

## 4 Concluding Remarks

EoP is a complex pathology with various effects on OL lineage which result in the different forms of WMD. On the one hand, pre-OL death occurs and, on the other hand, the pre-OL population increases due to an arrest of OL differentiation. Human data support that these two phenomena could coexist in a variable manner in the brain of infants suffering from EoP. Systemic inflammation is frequent during the perinatal period in very preterm infants. The systemic inflammation episodes can be multiple, varying in intensity and duration throughout this vulnerability window for the developing brain. Animal models confirm that systemic inflammation elicits the alterations of OL lineage observed in the EoP. Intriguingly, specific effects on OL lineage are observed depending on the systemic inflammation stimulus. Systemic LPS first induces Pre-OL death and an impairment of Pre-OL proliferation. The surviving proliferative OLs subsequently restore the complete OL lineage and myelination. Surprisingly, long-term trophic effects on the differentiated OLs sustaining the survival of mature OLs are induced by neonatal exposure to systemic LPS (Figure 1). In contrast, systemic IL-1β leads to an alteration of OL differentiation without any change in OL survival and proliferation (Figure 2). Thereafter, a progressive recovery of the OL differentiation is assumed with a catch-up of myelination. No prolonged action of systemic IL-1β on OL lineage has been identified. Hence, since both these stimuli result in delayed myelination, the underlying cellular mechanisms and the OL lineage trajectories are different and variable long-term consequences on the white matter may be speculated upon. Therefore, the quality and the density of myelin and the subsequent brain functioning may vary according to the history of the inflammatory challenges during the neonatal period. Furthermore, an earlier and more robust recovery of myelinating OLs as observed after systemic LPS could be associated with a more efficient rehabilitation of myelination. This hypothesis emerges from this review and needs further exploration.

In parallel, experimental data highlight the multiple cellular pathways that induce WMD in the inflammatory context. These data raise questions about the precise characterisation of the inflammatory stimuli in clinical practice in order to adapt neuroprotective and neurorepair strategies in preterm infants.
